# Hydrogen peroxide detection with high specificity in living cells and inflamed tissues

**DOI:** 10.1093/rb/rbw022

**Published:** 2016-06-11

**Authors:** Lei Rong, Chi Zhang, Qi Lei, Ming-Ming Hu, Jun Feng, Hong-Bing Shu, Yi Liu, Xian-Zheng Zhang

**Affiliations:** ^1^Department of Chemistry, Key Laboratory of Biomedical Polymers of Ministry of Education; ^2^State Key Laboratory of Virology, College of Life Sciences, Wuhan University, Wuhan, P. R. China

**Keywords:** hydrogen peroxide, fluorescence imaging, inflammation

## Abstract

Hydrogen peroxide (H_2_O_2_) detection in biological systems is of significant importance, which act as critical second messenger in fundamental biological processes. Here, we report on a chemoselective fluorescent naphthylimide peroxide probe (NPP) for the H_2_O_2_ detection in vitro and in vivo. NPP is a phenylboronic acid-caged chromophore that selectively responds to H_2_O_2_ through a self-immolate mechanism. NPP exhibited high sensitivity and selectivity to H_2_O_2_ with distinctive fluorescence change due to the excellent two-photon excitation property, which permits the facile detection of inflammation produced H_2_O_2_ and offers chance to monitor the inflammatory stages in diseased cells.

## Introduction

Reactive oxygen species (ROS) play essential roles in cellular physiological activities as signal transduction molecules [1]. The imbalance of ROS in cells is documented to be associated with many serious diseases, such as chronic inflammation [2], diabetes [3], Alzheimer’s disease[4, 5] and cancers [6]. Hydrogen peroxide (H_2_O_2_), a primary component in ROS family, acts as critical second messenger in fundamental biological processes [7]. The aberrant generation of H_2_O_2_ is a hallmark of oxidative stress and inflammatory reaction, which correlates closely with the onset and development of many serious diseases [3, 8–10]. For instance, evidences have shown that H_2_O_2_ is implicated in many inflammatory events, such as the activation of the proinflammatory cytokine tumour necrosis factor alpha (TNF-α) [11, 12]. The H_2_O_2_ concentration inside inflamed cells was identified to be dependent on the expression level of inflammatory genes and activation of inflammatory cytokines [11]. In this context, H_2_O_2_ detection in biological systems is of significant importance since it offers useful information to reflect the progression of many pathological events. To date, many fluorescent probes have been reported for cellular H_2_O_2_ detection [13]. For example, Wang *et al.* [14] reported an aggregation-induced emission-based fluorogenic probe for multianalyte detection including H_2_O_2_, and Abo *et al.* [15] used rhodamine based fluorogenic probe for specific H_2_O_2_ detection in living cells]. However, most reported probes operate in a nonspecific manner or barely meet the requirement of deep tissue imaging [16, 17].

**Figure 1.**
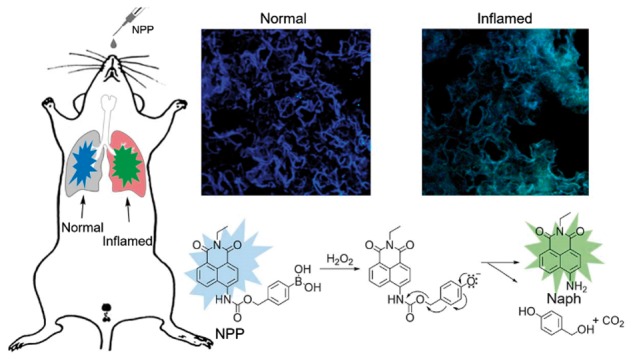
H_2_O_2_ detection in inflamed tissues and the possible mechanism for H_2_O_2_ induced fluorescence change.

In this study, we report a two-photon excited fluorescent H_2_O_2_ probe (naphthylimide peroxide probe, NPP) that could specifically respond to H_2_O_2_ in physiological conditions in an easy-to-monitor manner with deep tissue penetration capability [18–20]. In the design, H_2_O_2_ induced oxidation of boronic acid to phenolate would spontaneously cause an 1,6-elimination accompanied with the breakage of the self-immolative carbamate linker, leading to a fluorescently blue-to-green transition (Fig. 1) [21] with an ultrahigh signal-to-noise ratio [16, 22, 23]. *In vitro* and *in vivo* results show that the NPP is capable of specific detection on the inflammation produced H_2_O_2_ through two-photon imaging, offering chances to monitor the inflammatory stages in diseased cells.

## Experimental section

### Materials

The following materials and chemicals were used as received without further purification.

4-amino-1,8-naphthalic anhydride (95%), 4-(hydroxymethyl)phenylboronic acid pinacol ester (97%), lipopolysaccharides (LPS) from *Escherichia coli*, D-Penicillamine (DPA, US Pharmacopeia Reference Standard), catalase from bovine liver, and tert-butyl hydroperoxide solution (TBHP, 70 wt. % in H_2_O) were purchased from Sigma-Aldrich Co. LLC. (USA), used as received.

Triphosgene (99%), and 4-dimethylaminopyridine (99%) were purchased from Aladdin Industrial Inc. (China), and used as received.

Organic solvents and other chemicals used were provided by Shanghai Chemical Co. (China).

Dulbecco’s Modified Eagle Medium cell culture media, fetal bovine serum and phosphate buffered saline (PBS, pH = 7.4) were purchased from Life Technologies Corporation (USA), and used as recommended.

Trypsin-EDTA solution (0.05%, with phenol red) and Penicillin-Streptomycin solution (10 000 U/ml) were purchased from Biological Industries Israel Beit-Haemek Ltd. (Israel), and used as recommended.

### Synthesis of Naph

4-Amino-1,8-naphthalic anhydride (0.6 g) were dissolved in dimethylformamide (DMF) (15 ml), 2 ml ethylamine (65–70% aqueous solution) were added dropwise and the reaction mixture was stirred at 100°C overnight. The solution was cooled to room temperature and poured into ice-cold water (200 ml). The precipitate was filtered and washed with water, followed by ether, and dried over air. Yield 0.45 g, 67%. ^1^H-NMR (300 MHz, DMSO-d6) δ 8.55(d, 1H), 8.36(d, 1H), 8.13 (d, 1H), 7.60(t, 1H), 7.38(s, 2H), 6.77(d, 1H), 3.98(d, 2H), 1.12 (s, 3H).

### Synthesis of NPP-E

To a mixture of Naph (20 mg, 1 equiv) and DMAP (29.5 mg, 1 equiv) in dry toluene (3 ml) was added to a solution of triphosgene (24 mg, 1 equiv) in dry toluene slowly. The resulting solution was refluxed for 3 h. After cooling to room temperature, the reaction mixture was diluted with dry CH_2_Cl_2_ (about 6 ml) and filtered under reduced pressure. 4-(Hydroxymethyl)phenylboronic acid pinacol ester (18.7 mg, 1 equiv) were added to the filtrate and the solution was stirred at room temperature for an additional three hours. The reaction mixture was then concentrated and purified by flash column chromatography (silica gel, 20:1 chloroform/methanol) to give NPP-E as a yellow solid. Yield 34 mg, 83%. 1H-NMR (300 MHz, CDCl3) δ 8.55(d, 1H), 8.36(d, 1H), 8.13(d, 1H), 7.60(m, 3H), 7.38(m, 4H), 5.27(d, 2H), 4.20(d, 2H), 1.12(s, 15H).

### Synthesis of NPP

NPP-E (total from last step) was dissolved in 1 ml THF, followed by the addition of 7 ml of a 20% HCl aqueous solution. The yellowish slurry was stirred vigorously for 4 h at room temperature. The solids were centrifuged out and washed with ultrapure water four times, after which a little mount of water was added to the precipitates and the mixture was lyophilized to give NPP as a yellowish solid. Yield 24.7 mg, 87%. ^1^H-NMR (300 MHz, CD3OD) δ 8.58(m, 4H), 8.29(d, 1H), 7.83(t, 2H), 7.69(d, 2H), 7.51(d, 2H), 5.34(s, 2H), 4.23(m, 2H), 1.32(t, 3H) (see Supplementary Figure S1).

### Cellular and animal experiments

#### Ethics statement

Murine RAW 264.7 cells (mouse leukaemic monocyte macrophage cell line) were acquired from China Center for Type Culture Collection (Wuhan, China), and cultured with 1640 medium using standard protocol. Six- to eight-week old female C57BL/6 mice were acquired from Animal Experiment Center of Wuhan University (Wuhan, China), and fed with standard chow. All study protocols were approved by the Institutional Animal Care and Use Committee of the Animal Experiment Center of Wuhan University (Wuhan, China). All mouse experimental procedures were performed in accordance with the Regulations for the Administration of Affairs Concerning Experimental Animals approved by the State Council of People’s Republic of China.

#### LPS-induced H2O2 produce and fluorescence imaging

RAW 264.7 cells were seeded in Φ 20 mm glass bottom dishes (Corning, USA), and appropriate amount of LPS was added and incubated for the indicated time periods. After which, cells were labelled with NPP (final concentration 5 μM) and incubated at 37°C for another 40 min. The fluorescence images were obtained using two-photon laser scanning fluorescence microscopy (Zeiss NOL-LSM 710).

#### qPCR analysis of LPS-activated cells

RAW 264.7 cells were seeded in six-well plate (Corning, USA), and appropriate amount of LPS was added and incubated for the indicated time periods. After which, total RNA from RAW 264.7 cells was isolated using TRIzol reagent (Invitrogen, The Netherlands). After reverse transcription with oligo (dT) primer using a RevertAidTM First Strand cDNA Synthesis Kit (Fermentas, *U*SA), aliquots of products were subjected to real-time PCR analysis to measure mRNA expression levels of tested genes. GAPDH was used as a reference gene. Gene-specific primer sequences were the following:

5′-ACGGCCGCATCTTCTTGTGCA-3′ (GAPDH forward),

5′-ACGGCCAAATCCGTTCACACC-3′ (GAPDH reverse);

5′-GGTGATCGGTCCCCAAAGGGATGA-3′ (TNF-α forward),

5′-TGGTTTGCTACGACGTGGGCT-3′ (TNF-α reverse);

5′-CGGACCCCAAAAGATGAAGGGCTG-3′ (IL-1β forward),

5′-AGCTGCCACAGCTTCTCCACA-3′ (IL-1β reverse);

5′-TCTGCAAGAGACTTCCATCCAGTTGC-3′ (IL-6 forward),

5′-AGCCTCCGACTTGTGAAGTGGT-3′ (IL-6 reverse).

#### Quantitative cellular H2O2 concentration detection in living cells

RAW 264.7 cells were seeded in 96-well plates, LPS (1 μg/ml) was added and incubated for the indicated time periods. After which, the cellular H_2_O_2_ concentration was detected with Amplite Fluorimetric Hydrogen Peroxide Assay Kit (AAT Bioquest, Inc., CA) using recommended protocol.

#### LPS-induced lung inflammation and imaging

Six- to eight-week old female C57BL/6 mice (Animal Experiment Center of Wuhan University, Wuhan, China) were administrated with 20 μg LPS in 35 μl of PBS intranasally (i.n.) on four consecutive days. 24 h after the final i.n. challenge with LPS, the mice were i.n. administrated with NPP (10 nmol in aqueous solution). 40 min later, the mice were euthanized and lungs were harvested, frozen-sectioned at a thickness of 10 μm, mounted on slides, and visualized by two-photon laser scanning fluorescence microscopy (Olympus FV1200MPE). For the mice treated with DPA, the DPA (1 μmol) were i.n. administrated 5 min prior to NPP administration. Age- and sex-matched control mice were also tested.

#### H&E staining

Lung sections (10 μm) were cut and stained with hematoxylin and eosin to verify lung inflammation in the LPS model.

#### LPS-induced rear paw inflammation and imaging

Six- to eight-week old female C57BL/6 mice were injected with 20 μg LPS on the right rear paw after anesthesia. After 1 day, NPP (10 nmol) were injected subcutaneously on the right and left rear paw. 1 h later, the mice were euthanized and the skin of the inflamed (right paw) and normal (left paw) tissue were harvested, frozen-sectioned at a thickness of 10 μm, mounted on slides, and visualized by two-photon laser scanning fluorescence microscopy (Nikon A1).

## Results and discussion

**Figure 2.**
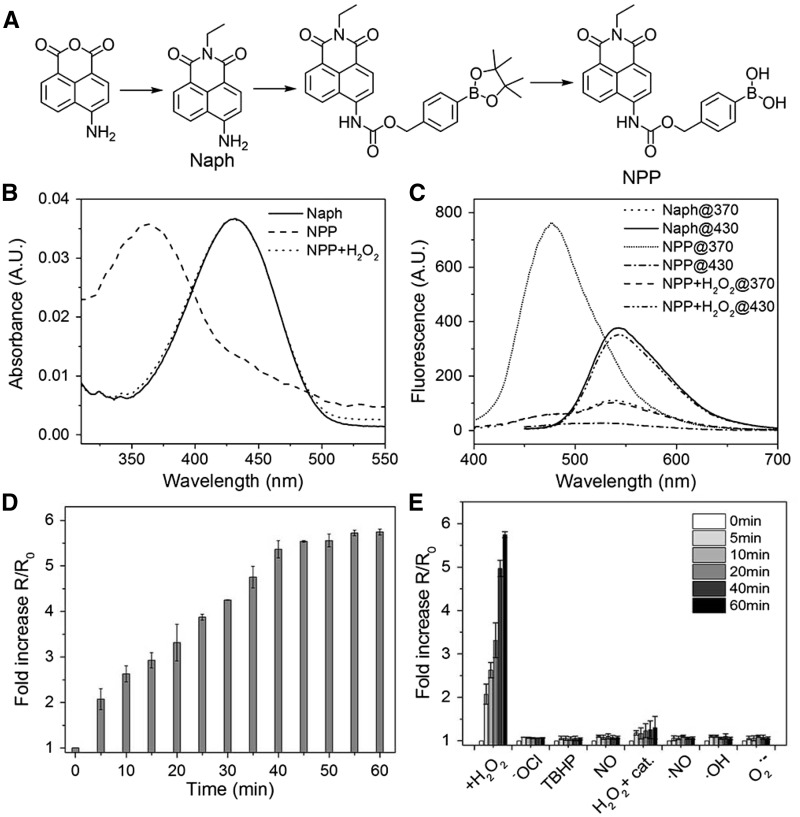
(A) Synthesis of NPP. **(B)** UV absorption of NPP before and after exposed to H_2_O_2_, and Naph. **(C)** Fluorescence emission of NPP before and after exposed to H_2_O_2_, and Naph. **(D)** Fold increase in green fluorescence intensity of NPP (5 µM) incubated with H_2_O_2_ (100 µM) within 60 min, fluorescent data were acquired every 5 min (excitation at 430 nm). **(E)** Fold increase in green fluorescence intensity of NPP (5 µM) incubated with indicated ROS or their donors (100 µM; the catalase concentration, when present, were 0.5 mg/ml; excitation at 430 nm). All spectra, if not indicated specially, were acquired 60 min after the addition of indicated ROS under simulated physiological conditions (37 °C in PBS buffer, pH 7.4). TBHP, tert-butyl hydroperoxide.

The NPP was prepared by the synthetic route outlined in Figure 2A. The pinacol ester was removed for better H_2_O_2_ sensitivity and water solubility [24]. The chemical structure of NPP were confirmed using ^1^H-NMR, ^13^C-NMR and Electrospray Ionization Mass Spectrometry (see Supplementary Figure S1). The fluorescence property of NPP was studied in simulated physiological conditions (PBS, pH 7.4, 37°C). As shown in Figure 2C, in the absence of H_2_O_2_, NPP demonstrated strong emission at 460 nm (blue); after exposed to the H_2_O_2_, the emission band red-shifted to 540 nm (green). This spectral change was initiated by the H_2_O_2_-induced self-immolative elimination of the phenylboronic acid moiety, which changed the blue fluorescent NPP into the green fluorescent Naph (Fig. 1). This strong band shift of emission (80 nm) ensured the good signal-to-noise ratio in the following H_2_O_2_ detection. In addition, the self-immolative elimination based fluorescence change mechanism was confirmed by the fully consistent of the fluorescence spectra and UV absorbance spectra of H_2_O_2_-exposed NPP and Naph (Fig. 2B and C) [21, 22].

Next, the selectivity of the NPP to H_2_O_2_ over other biologically relevant cellular ROS was evaluated. NPP were incubated with various ROS, and the fold increase in green fluorescence intensity of NPP before and after exposed to these ROS was monitored for 60 min. When NPP exposed to H_2_O_2_, a time-dependent increase was observed, and it was found to reach a plateau after incubated with H_2_O_2_ for 40 min (Fig. 2D). In addition, chemical kinetic modeling shows that, within 30 min, the reaction between NPP and H_2_O_2_ follows a second-order kinetics, with *k* = 0.05557 M^−1^s^−1^ (see Supplementary Figure S2A–E). Moreover, a linear relationship (*R*^2 ^=^ ^0.998) between the fluorescence intensity and the H_2_O_2_ concentration was observed (5 µM NPP were used. See Supplementary Figure S2F). In contrast, other biologically relevant cellular ROS had little to no effect on the green fluorescence increasing (Fig. 2E). With the presence of catalase, a selective H_2_O_2_ scavenger, negligible fold increase of the green fluorescence was observed (Fig. 2E, H2O_2_ +catalase). These findings verified the robust and specific H_2_O_2_-induced fluorescence change of NPP. Moreover, NPP and Naph showed high two-photon action cross section value (49 GM at 710 nm and 35 GM at 810 nm, respectively.), which suggested excellent two-photon excitation capability in simulated physiological conditions (see Supplementary Figure S3). Overall, the fluorescent properties observed above established that NPP could selectively react with H_2_O_2_ in physiological conditions in a concentration dependent manner with the optimal reaction time of 40 min, and hold the merit of two-photon excitation.

**Figure 3.**
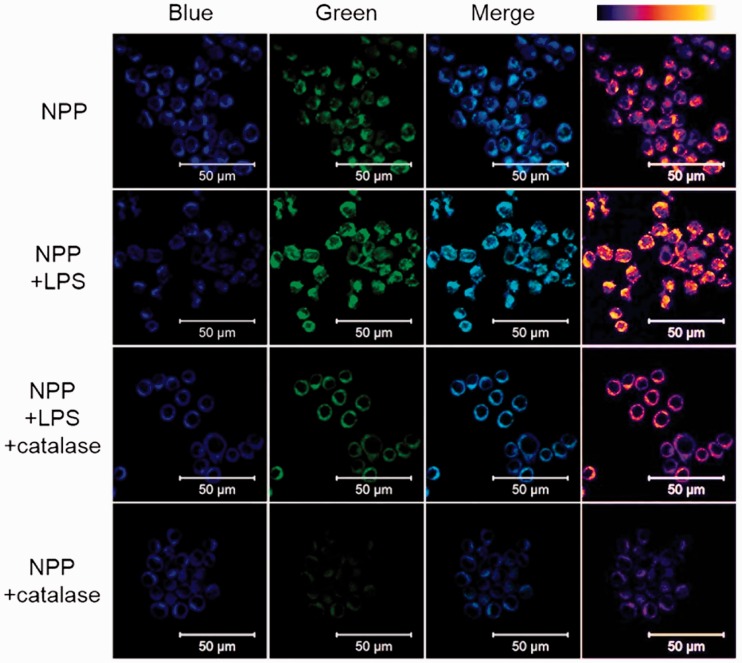
Fluorescence images of RAW 264.7 cells labelled by NPP under indicated conditions. NPP, cells without LPS or catalase treatment; NPP +LPS, cells treated with LPS; NPP +LPS +catalase, cells treated with both LPS and catalase; NPP +catalase, cells treated with catalase. Images were observed using two-photon laser scanning fluorescence microscopy (NPP, 5 µM; LPS, 1 µg/ml; excited at 740 and 810 nm for blue and green fluorescence, respectively). False colour image of green channel (right line) was acquired using ImageJ. Scale bar: 50 µm.

Based on the findings above, NPP was applied for the cellular H_2_O_2_ detection in inflamed cells. Murine RAW 264.7 macrophages were used as cellular model. LPS, a strong inflammation stimulator, was employed for inflammatory response. When treated with LPS, cells demonstrated bright fluorescence in green (Fig. 3, NPP + LPS). And due to the sensitivity of the NPP, the H_2_O_2_ in normal cells (0–5 μM, generated by respiration) was also be detected and result in faint green fluorescence (Fig. 3, NPP) [25–28]. This dramatic fluorescence change was attributed to the presence of LPS, which caused acute inflammatory response accompanied with the production of cellular H_2_O_2_ [29]. In contrast, when cellular H_2_O_2_ in LPS-treated cells were eliminated with catalase, the green fluorescence dimmed apparently compared with LPS-treated cells, and normal cells. This sharp decrease was ascribed to the excessive catalase added, which eliminated the H_2_O_2_ generated by inflammatory response and respiration. In addition, cells treated with catalase only showed the weakest green fluorescence, which confirmed the H_2_O_2_-specific fluorescence change in cells. These results suggested that NPP could specifically detect cellular H_2_O_2_ in a concentration dependent manner, and hold the potential to reveal the inflammatory status in macrophage cells.

**Figure 4.**
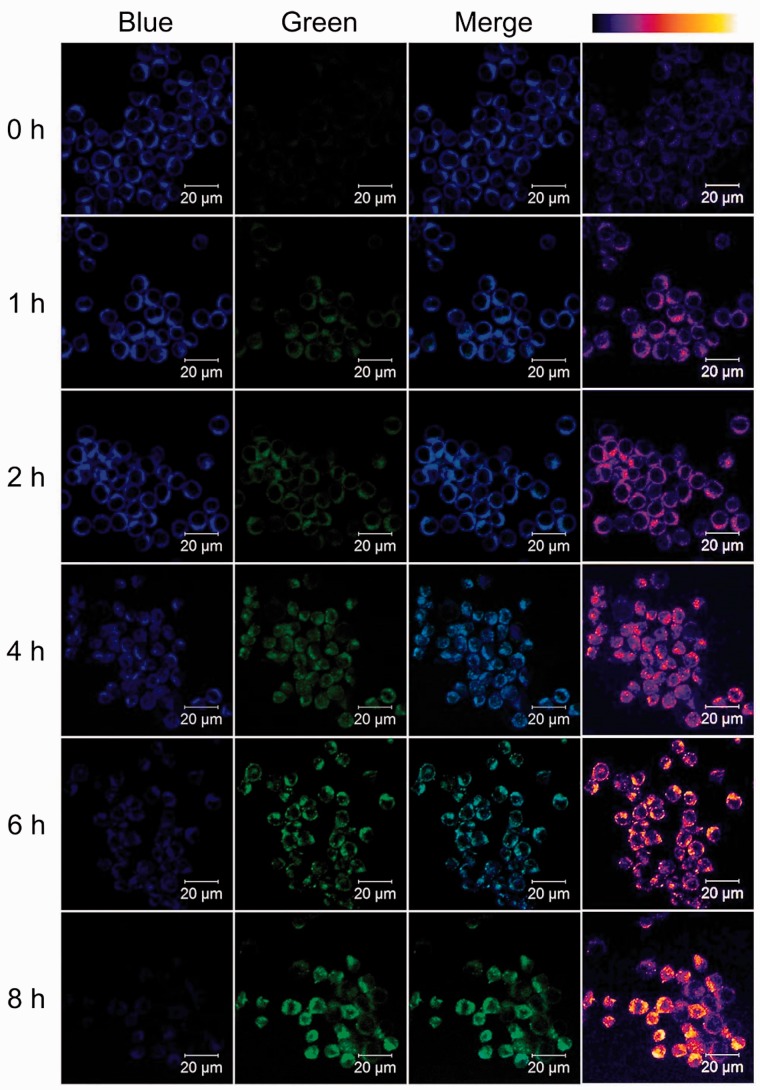
Fluorescent images of RAW 264.7 cells with the presence of LPS (1 µg/ml) for indicated time periods. Images were observed using two-photon laser scanning fluorescence microscopy (NPP, 5 µM; excited at 740 and 810 nm for blue and green fluorescence, respectively). False colour image of green channel (right line) was acquired using ImageJ. Scale bar: 20 µm.

**Figure 5.**
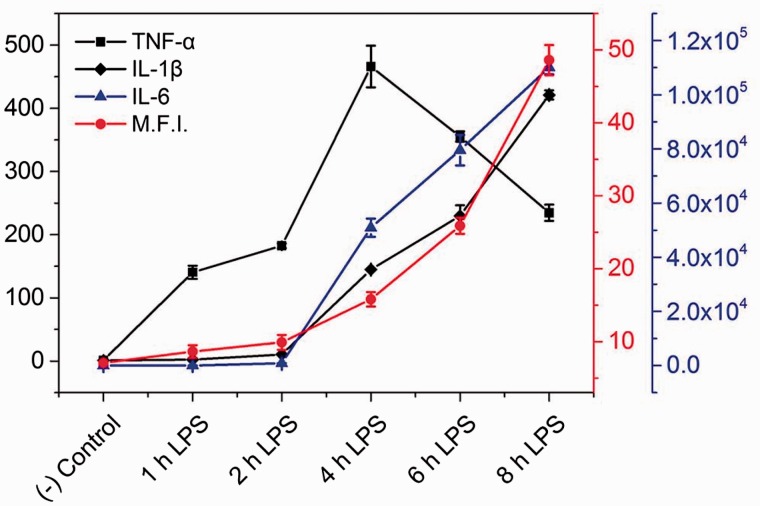
Trends of green fluorescence change and inflammation related mRNA levels in RAW 264.7 cells with the presence of LPS (1 µg/ml) for indicated periods. (−) Control refer to the RAW 264.7 cells without LPS treatment.

In order to confirm the cellular inflammatory status detection capability of the NPP, RAW 264.7 cells were treated with LPS for different time periods ranging from 0 to 8 h. As shown in Figure 4, with the culturing time increases, the green fluorescence increased gradually along with the decrease of the blue fluorescence. This fluorescence change was ascribed to the gradually increased inflammatory response caused by LPS, which results in the time-depended increase of the H_2_O_2_ concentration in RAW 264.7 cells. As inflammatory response started, the expression of several proinflammatory NF-κB-dependent genes induced by TNF-α were increased, such as TNF-α, interleukin-1β (IL-1β), and IL-8 [11]. Meanwhile, some anti-inflammatory gene expression were also increased, such as IL-6.11 Thus, RAW 264.7 cells treated with LPS for different time periods were performed with qPCR (quantitative polymerase chain reaction) analysis, and the transcription of TNF-α, IL-1β and IL-6 genes were detected. As an inducer of the cytokine IL-1β, the expression of TNF-α gene increased first and reached the top peak value at 4 h, then start to decrease. Meanwhile, the TNF-α-induced proinflammatory gene IL-1β and anti-inflammatory gene IL-6 were stated to increase 2 h after the addition of the LPS (see Supplementary Figure 4A). The increase of gene levels in these genes indicated the increased level of TNF-α, IL-1β and IL-6 cytokines, which confirmed the LPS-induced time-dependent inflammatory response in RAW 264.7 cells. The mean fluorescence intensity (M.F.I.) of green fluorescence in Figure 4 was acquired using ImageJ. As shown in Figure 5, the trend of green fluorescence change was consisting very well with the trends of inflammation associated cytokine gene levels. Similar results were also observed in TNF-α and IL-6 expression by ELISA analysis (see Supplementary Figure S4B), and cellular H_2_O_2_ concentration in LPS-treated cells (see Supplementary Figure S4C). These findings confirmed that NPP holds the capability of revealing inflammatory status in living cells with a significant fluorescence change.

**Figure 6.**
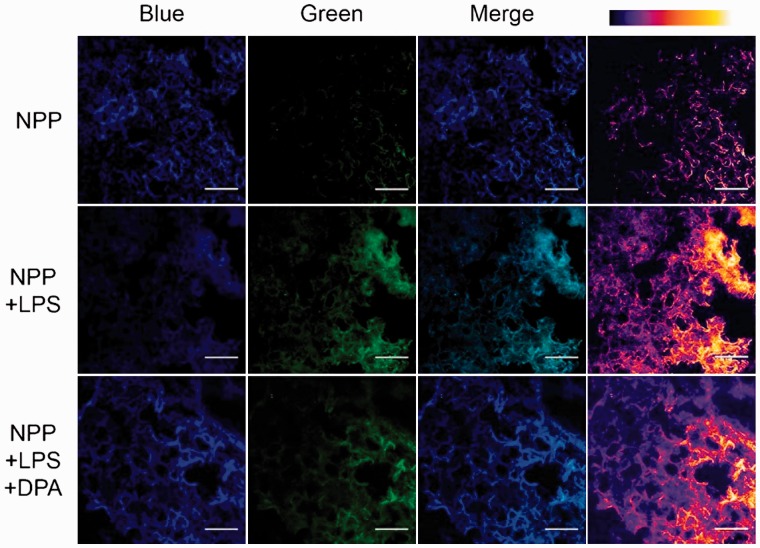
Detection of LPS-induced H_2_O_2_ produced in inflammatory lungs using NPP. NPP, mice without LPS or DPA treatment, mice treated with LPS; NPP + LPS + DPA, mice treated with LPS and DPA. Images were observed using two-photon laser scanning fluorescence microscopy (excited at 740 and 810 nm for blue and green fluorescence, respectively.). False colour image of green channel (right line) was acquired using ImageJ. Scale bar: 100 μm.

Next, NPP was applied for the detection of H_2_O_2_ produced endogenously by lung inflammation in model animals. LPS was used for the activation of inflammatory response [30]. To this end, C57BL/6 mice were either treated or not with 20 μg LPS by intranasal (i.n.) administration for 4 consecutive days [31]. 40 min after NPP (10 nM) i.n. administrated, the mice were euthanized and lungs were harvested, frozen-sectioned at a thickness of 10 μm, mounted on slides, and visualized (Fig. 6, top and middle row). Recruitment of inflammatory cells to the airways of LPS-treated mice was confirmed by hematoxylin and eosin staining (see Supplementary Figure S5). Moreover, LPS-treated mice were given DPA (1 μM), a H_2_O_2_ scavenger [32], 5 min prior to NPP i.n. administration, which were used as reference (Fig. 6, bottom row). As shown in Figure 6, obvious blue-to-green fluorescence change was observed in the lungs of LPS-treated mice while negligible fluorescence changes were found in lungs of mice without LPS treatment. This fluorescence difference was attributed to the increased cellular H_2_O_2_ endogenously produced by activated macrophages and neutrophils, which were recruited to the airways of the mice due to the LPS-induced inflammation. When compared with the lungs of LPS-treated mice, lungs of mice treated with both LPS and DPA showed much weaker fluorescence change (Fig. 6, bottom row). In this case, the DPA administrated efficiently eliminated the H_2_O_2_ produced by the inflamed tissues and led to the weaker fluorescence change observed. This DPA-caused difference confirmed that NPP could specifically response to the H_2_O_2_ in inflamed tissues. Moreover, NPP also held the capability of detecting H_2_O_2_ produced in inflamed skins in paw-inflamed mouse model (see Supplementary Figure S6). Taken together, these findings suggested that NPP could specifically respond to endogenous levels of H_2_O_2_ produced in inflammation *in vivo*.

## Conclusions

In summary, The NPP could react selectively and in a concentration-dependent manner with H_2_O_2_ in physiological conditions in an easy-to-monitor manner. These properties enabled NPP for the detection of cellular H_2_O_2_ produced by inflamed macrophage cells, and could reveal the inflammatory status with remarkable fluorescence change. Moreover, NPP was sensitive enough to detect H_2_O_2_ endogenously produced by inflamed tissues *in vivo*. The development of NPP could potentially enable the fast *in situ* inflammatory response detection in living systems. Importantly, similar strategies could also be used for the fast detection of other biomolecules in biological environments.

## Supplementary data

Supplementary data is available at *REGBIO* online.

## Funding

This work was supported by the National Natural Science Foundation of China (21474077, 51233003 and 51533006) and the Fundamental Research Funds for the Central Universities.

*Conflict of interest statement*. None declared.

## Supplementary Material

Supplementary Figure S1
