# Supplementary material for: Hydrogen peroxide detection with high specificity in living cells and inflamed tissues
Source: Regen Biomater. 2016 Jun 11;3(4):217–22. doi: 10.1093/rb/rbw022 (PMC4966294; doi:10.1093/rb/rbw022)
Supplement: Supplementary Figure S1 [file rb_rbw022_index.html]

Supplementary Data | Regenerative Biomaterials

## Supplementary Data

files

- Supplementary Data - doc file
