# Supplementary material for: Hydrogen peroxide detection with high specificity in living cells and inflamed tissues
Source: Regen Biomater. 2016 Jun 11;3(4):217–22. doi: 10.1093/rb/rbw022 (PMC4966294; doi:10.1093/rb/rbw022)
Supplement: Supplementary Figure S1 [file Supporting_Information_R.doc]

**Supporting Information**

**Hydrogen peroxide detection with high specificity in living cells and inflamed tissues**

Lei Rong,^1,2^ Chi Zhang,^1^ Qi Lei,^1^ Ming-Ming Hu,^2^ Jun Feng,^1^ Hong-Bing Shu,^2^ Yi Liu,^1^ and Xian-Zheng Zhang^1^,*

^1^ Key Laboratory of Biomedical Polymers of Ministry of Education & Department of Chemistry, Wuhan University, Wuhan, P. R. China; ^2^ State Key Laboratory of Virology, College of Life Sciences, Wuhan University, Wuhan, P. R. China

*Correspondence address. Key Laboratory of Biomedical Polymers of Ministry of Education & Department of Chemistry, Wuhan University, Wuhan 430072, China. Tel: +86-27-68755993; Fax: +86-27-68755993; E-mail: xz-zhang@whu.edu.cn

**Supportive figures**

**Supplementary Scheme 1.** Synthesis routes of NPP.


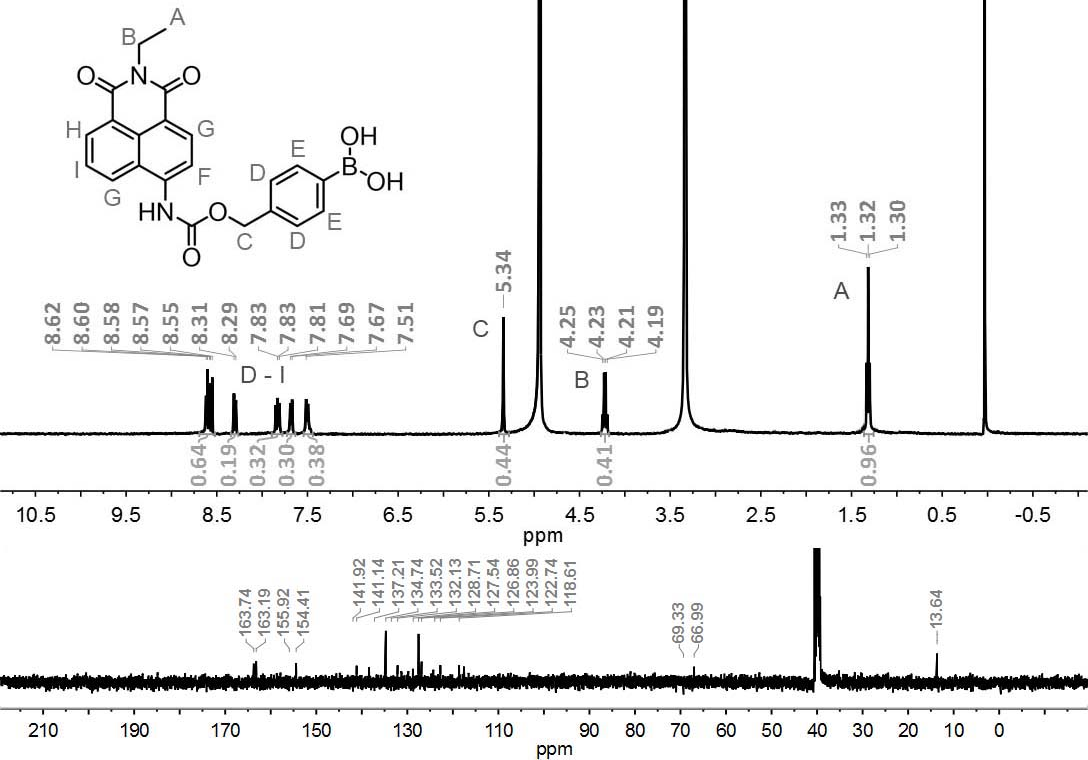


**Supplementary Figure 1**. ^1^H-NMR and ^13^C-NMR spectrum of NPP


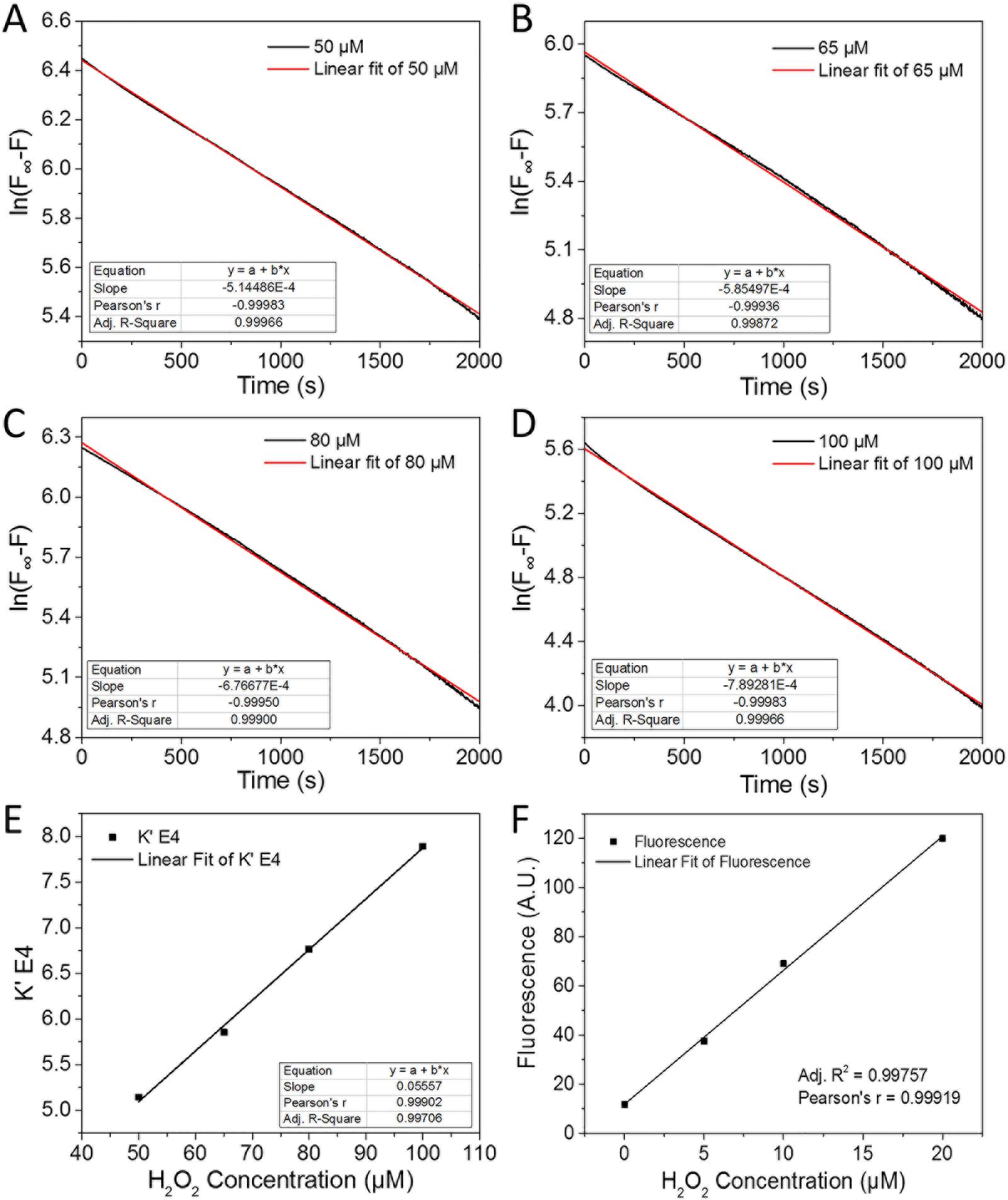


**Supplementary Figure 2**. (A-D) Plot of ln(F_∞_-F) against time at 540 nm, where F_∞_ and F are the fluorescence intensities at 540 nm at t_∞_ (= 40 min) ant t, respectively. NPP (5 µM) incubated with H_2_O_2_ at indicated concentrations for 33 min, and the fluorescence intensity of NPP solution was measured every second (excitation at 430 nm, emission at 540 nm). (E) Measurement of second-order rate constant for the reaction of NPP with H_2_O_2_. Values of K’ were from A-D. (F) Linear relationship (R^2^ = 0.998) between the fluorescence intensity at 540 nm and the H_2_O_2_ concentration (0 - 20 μM). 5 μM NPP were incubated with H_2_O_2_ (0, 5, 10, and 20 μM) under simulated physiological conditions. The H_2_O_2_ concentration in living cells is believed to be below 20 μM.^[S1, S2]^


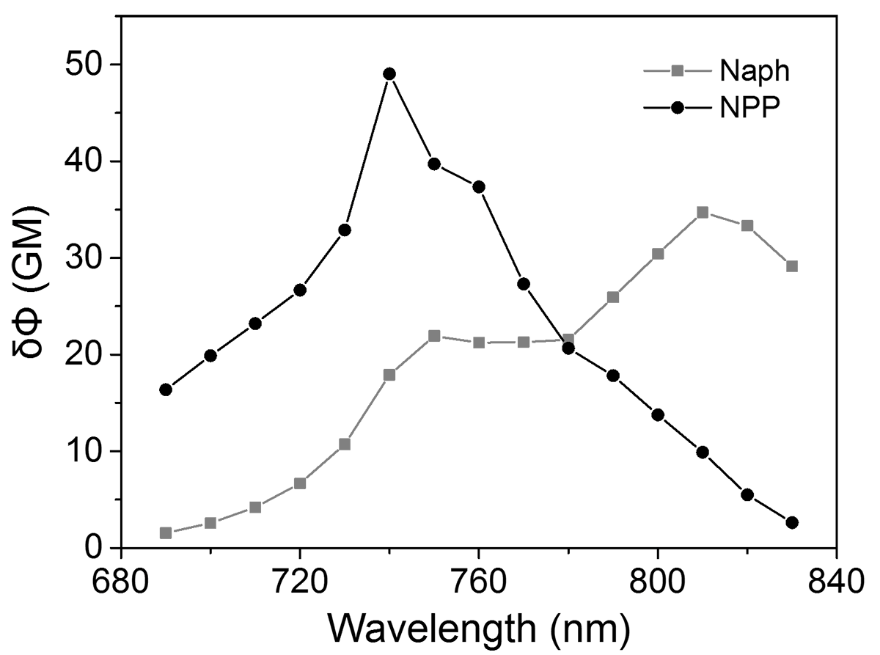


**Supplementary Figure 3**. Two-photon action spectra of 5 μM Naph and 5 μM NPP in PBS buffer (pH 7.4, 37 °C). The estimated uncertainties for the two-photon action cross section values (Φδ) are ±15%. The maximum two-photon action cross section value (Φδmax) of NPP and Naph were found to be 49 GM at 740 nm and 35 GM at 810 nm, respectively. These findings indicated that NPP could be used as a proper two-photon excitation probe for H_2_O_2_ in physiological conditions.


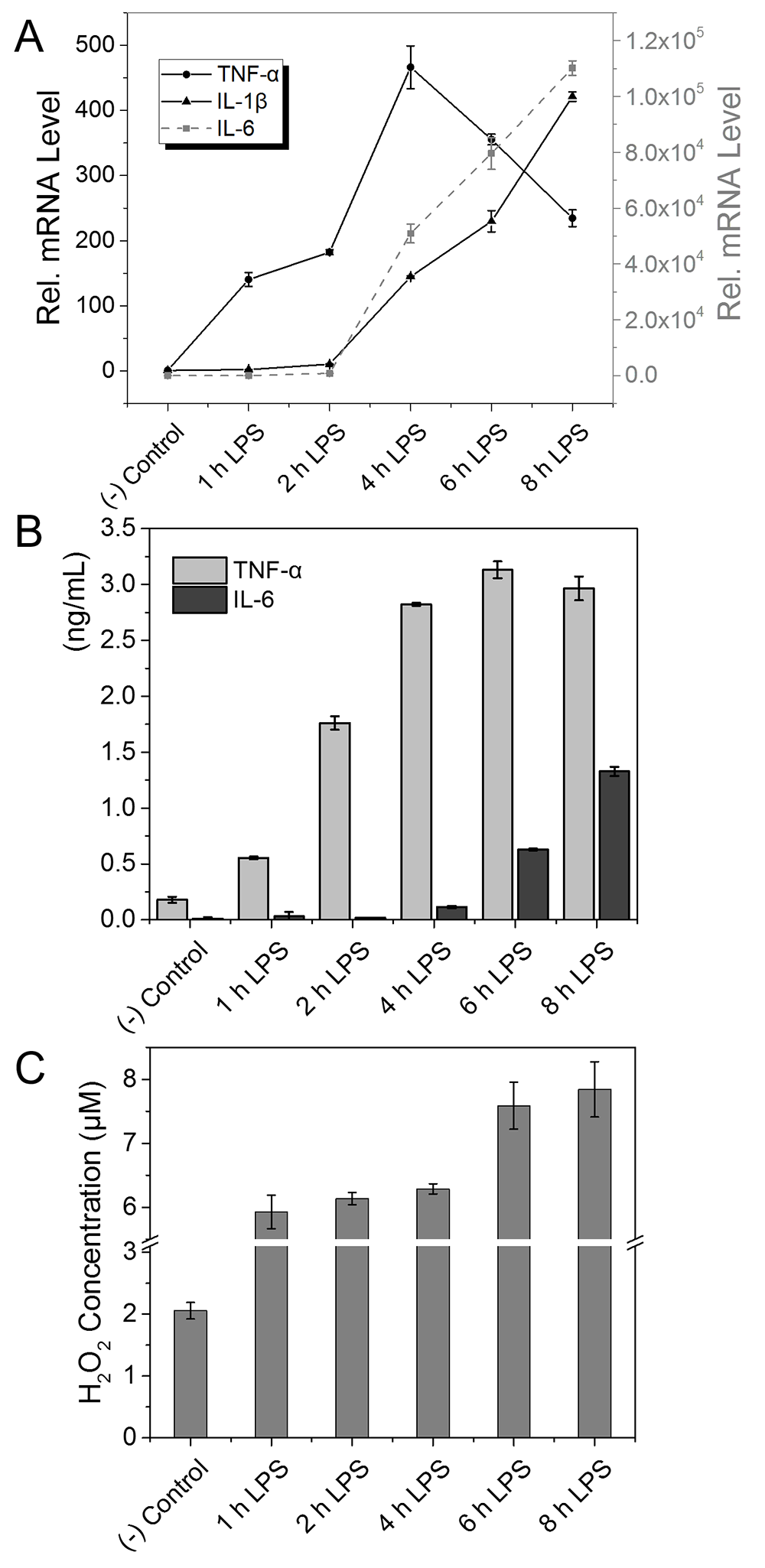


**Supplementary Figure 4**. (A) LPS-induced transcription of TNF-α, IL-1β, and IL-6 genes of RAW 264.7 cells with the presence of LPS (1 μg/mL) for indicated time periods. RAW 264.7 cells were treated with LPS (1 μg/mL) for indicated time periods, after which, total RNA was prepared for qPCR analysis. (B) LPS-induced cytokine of TNF-α, and IL-6 in RAW 264.7 cells with the presence of LPS (1 μg/mL) for indicated time periods. RAW 264.7 cells were treated with LPS (1 μg/mL) for indicated time periods, after which, the medium was collected for ELISA analysis. (C) H_2_O_2_ concentration in RAW 264.7 cells with the presence of LPS (1 μg/mL) for indicated time periods. The H_2_O_2_ concentration was quantitatively detected using a commercial assay kit. (-) Control refer to the RAW 264.7 cells without LPS treatment.


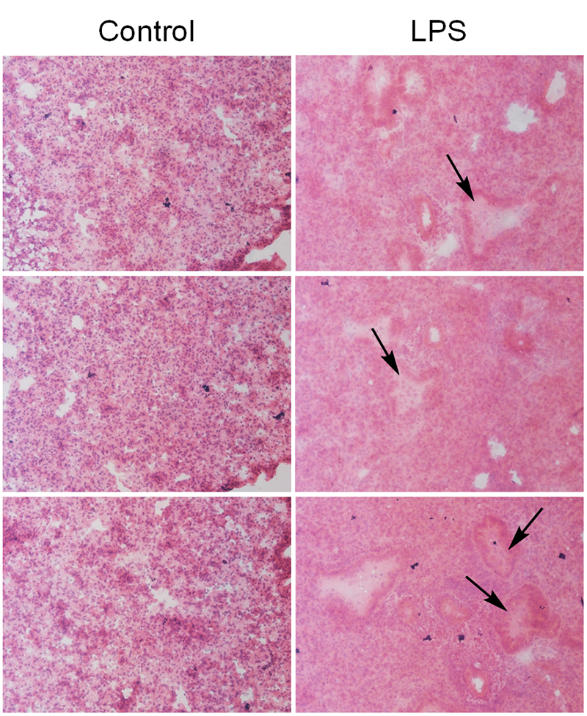


**Supplementary Figure 5**. Hematoxylin and eosin (H&E) staining of mice treated with (LPS) or without (Control) LPS shows recruitment of inflammatory cells to the airways of mice treated with LPS. Black arrows indicated the recruited inflammatory cells in the airways.


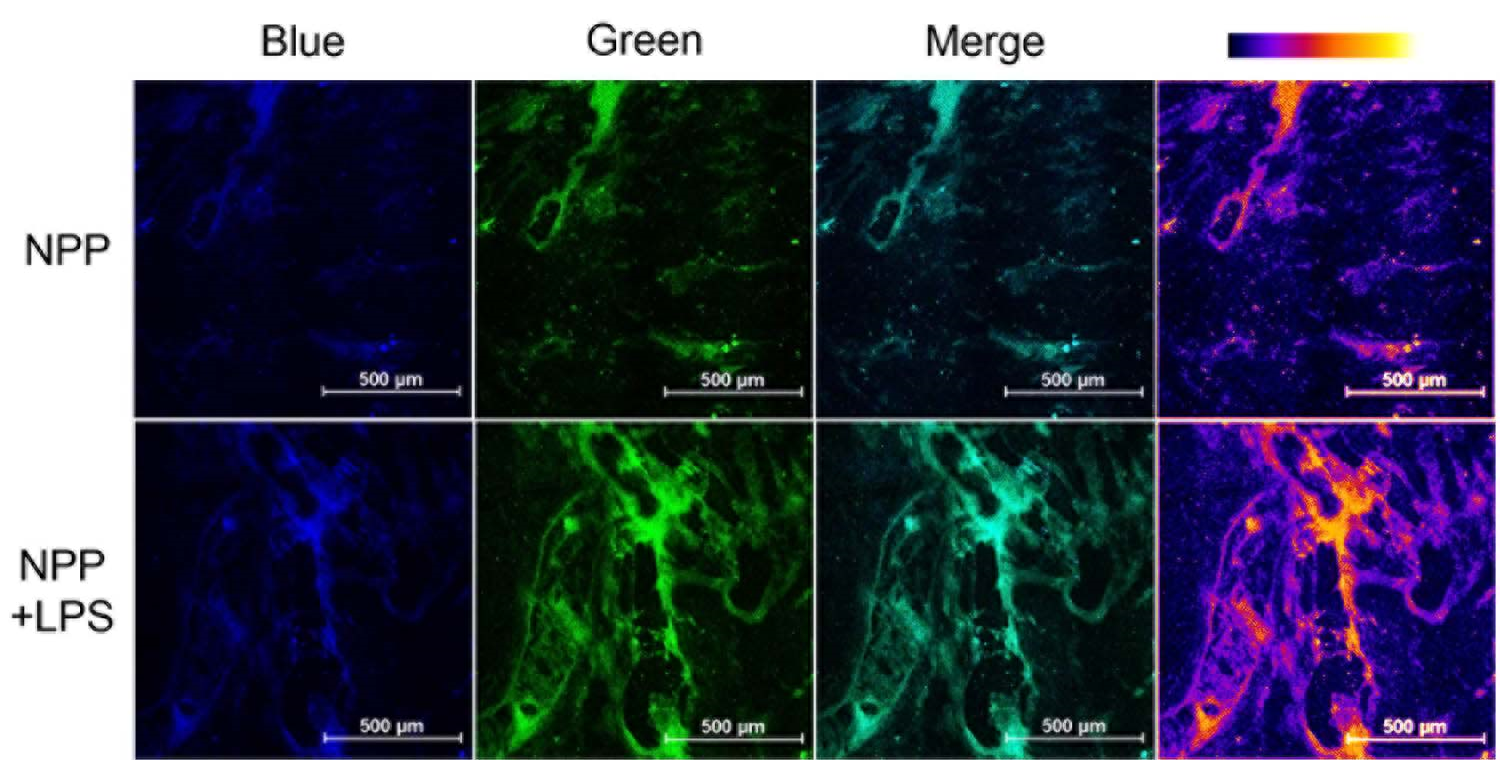


**Supplementary Figure 6**. Detection of LPS-induced H_2_O_2_ produced in inflammatory rear paws of mouse using NPP. NPP, mice without LPS treatment and labelled with NPP; NPP+LPS, mice treated with LPS and labelled with NPP. Images were observed using two-photon laser scanning fluorescence microscopy (excited at 740 nm and 810 nm for blue and green fluorescence, respectively.). False color image of green channel (right line) was acquired using ImageJ. Scale bar: 500 µm.

**References**

[S1] de Oliveira-Marques, V.; Cyrne, L.; Marinho, H. S.; Antunes, F. A quantitative study of NF-κB activation by H2O2: relevance in inflammation and synergy with TNF-α. *J. Immunol.,* **2007**, 178(6): 3893-3902.

[S2] Oliveira-Marques, V.; Marinho, H. S.; Cyrne, L.; Antunes, F. Role of hydrogen peroxide in NF-κB activation: from inducer to modulator. *Antioxid. Redox Signaling*, **2009**, 11(9): 2223-2243.
